# Microclimate predicts frost hardiness of alpine *Arabidopsis thaliana* populations better than elevation

**DOI:** 10.1002/ece3.5659

**Published:** 2019-10-09

**Authors:** Christian Lampei, Jörg Wunder, Thomas Wilhalm, Karl J. Schmid

**Affiliations:** ^1^ Institute of Plant Breeding, Seed Science and Population Genetics University of Hohenheim Stuttgart Germany; ^2^ Institute of Landscapes Ecology University of Münster Münster Germany; ^3^ Max Planck Institute for Plant Breeding Research Köln Germany; ^4^ Museum of Nature South Tyrol Bozen Italy

**Keywords:** altitude, *Arabidopsis thaliana*, common garden, elevation, frost hardiness, local adaptation, microclimate

## Abstract

In mountain regions, topological differences on the microscale can strongly affect microclimate and may counteract the average effects of elevation, such as decreasing temperatures. While these interactions are well understood, their effect on plant adaptation is understudied.

We investigated winter frost hardiness of *Arabidopsis thaliana* accessions originating from 13 sites along altitudinal gradients in the Southern Alps during three winters on an experimental field station on the Swabian Jura and compared levels of frost damage with the observed number of frost days and the lowest temperature in eight collection sites.

We found that frost hardiness increased with elevation in a log‐linear fashion. This is consistent with adaptation to a higher frequency of frost conditions, but also indicates a decreasing rate of change in frost hardiness with increasing elevation. Moreover, the number of frost days measured with temperature loggers at the collection sites correlated much better with frost hardiness than the elevation of collection sites, suggesting that populations were adapted to their local microclimate. Notably, the variance in frost days across sites increased exponentially with elevation. Together, our results suggest that strong microclimate heterogeneity of high alpine environments can preserve functional genetic diversity among small populations.

Synthesis: Here, we tested how plant populations differed in their adaptation to frost exposure along an elevation gradient and whether microsite temperatures improve the prediction of frost hardiness. We found that local temperatures, particularly the number of frost days, are a better predictor of the frost hardiness of plants than elevation. This reflects a substantial variance in frost frequency between sites at similar high elevations. We conclude that high mountain regions harbor microsites that differ in their local microclimate and thereby can preserve a high functional genetic diversity among them. Therefore, high mountain regions have the potential to function as a refugium in times of global change.

## Graphical abstract

Natural populations of *Arabidopsis thaliana* collected along an altitudinal gradient in the Southern Alps were studied regarding their adaptation to frost exposure. We tested the hypothesis that microsite temperatures are a better predictor for frost hardiness than elevation. We found that small scale local temperatures, particularly the derived number of frost days, predict frost hardiness better in the high mountains. This is due to the fact that high elevation sites differed strongly in frost frequency. These results indicate that high mountains offer heterogeneous microsites which can preserve a high functional genetic diversity between small populations which, in turn, have the potential to function as refuges in times of global climate change.

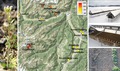

## 1 INTRODUCTION

In sessile organisms like plants, local adaptation along environmental gradients, such as aridity or temperature gradients, may lead to clinal trait variation. For example, seed dormancy increases linearly with decreasing rainfall in many annual dryland species (Hacker, 1984; Hacker & Ratcliff, 1989; Kronholm, Picó, Alonso‐Blanco, Goudet, & De, 2012; Lampei, Metz, & Tielbörger, 2017; Tielbörger, Petruů, & Lampei, 2012; Volis, Mendlinger, & Ward, 2002; Wagmann et al., 2012). Mostly, environmental gradients result from geographical or geological conditions (e.g., elevation differences or rain shadow) that influence local climatic conditions. However, while geography may cause gradual changes in environmental variables at large spatial scale, microtopography may confound it at small spatial scale, resulting in a heterogeneous microclimate landscape. Exposure, snow cover, soil type, and soil depth are just a few potential confounding factors which often vary on a local scale. For these reasons, geographical gradients such as latitudinal or elevational gradients are often poor proxies for the continuous change in single environmental factors (De Frenne et al., 2013; Graae et al., 2012; Körner, 2007).

Effects of microtopography may severely complicate the modeling of climate change effects for the prediction of species distribution ranges especially for high‐elevation sites (Dobrowski, 2011; Graae et al., 2012; Oldfather & Ackerly, 2019). This is because, on average high mountain sites vary stronger in microtopographic factors, such as aspect, inclination, and exposedness, than sites at lower elevation. For example, in high mountains, cold air drainage causes exposed sites to be warmer and couloirs or high mountain valleys to be colder than expected from interpolations across weather stations (Dobrowski, 2011; Graae et al., 2012). Consequently, migration to higher elevation may not be sufficient to follow the climatic niche because top‐soil temperatures may be decoupled from atmospheric air temperatures. On the other hand, a heterogeneous microclimate landscape may allow species to follow their climatic niche by migrating just “around the corner” (i.e., change exposure) instead of moving at larger geographical scales. Microclimate variation is not restricted to elevation gradients, but creates heterogeneity across all kinds of environmental gradients, such as rural‐urban gradients, latitudinal or aridity gradients. At the edge of the climatic niche of a species, microtopographic heterogeneity along gradients may provide islands with suitable microclimate for survival. Therefore, understanding how microclimate changes along environmental gradients and how this affects the adaptation and distribution of plants is an important task. However, while the general effects of topography on microclimate are well described based on various types of sensory data (Dobrowski, 2011; Graae et al., 2012; Scherrer & Körner, 2009, 2011), surprisingly few studies are available that investigated the effects of microclimate on plant adaptation in mountainous regions.

One factor that changes with higher altitude is temperature. On average, atmospheric temperature drops by 5.5°C per 1,000 m altitude (Körner, 2007). This results in a shift of growing season with vegetative growth starting later at higher elevations, which partly offsets the average temperature difference for plants. Nevertheless, at high elevations frequent and quick weather changes may lead to sudden frost periods. This explains why the ability to survive frost events is a typical adaptation to climate at higher elevations (Sakai & Otsuka, 1970). For instance, in the central European Alps species with a higher upper distribution boundary showed increased summer frost resistance (Taschler & Neuner, 2004). Also, Andean forbs and grasses from a high‐elevation site at 3,600 m showed higher frost resistance than conspecifics from a lower site at 2,800 m (Sierra‐Almeida, Cavieres, & Bravo, 2009). However, higher elevation populations are not always more resistant to frost. In Sweden, *Pinus sylvestris* showed higher frost resistance with higher latitude, but not with higher elevation (Sundblad & Andersson, 1995). We previously showed that Southern Alpine *Arabidopsis thaliana* populations from 2,200 to 2,350 m were not better adapted to frost experience than valley populations from 600 to 1,000 m elevation despite significant variation in frost hardiness among populations (Günther, Lampei, Barilar, & Schmid, 2016). In this study, we argued that microclimatic effects together with a delayed start of the growing season may reduce the risk of frost damage at higher elevation sites. According to the “law of the relative constancy of habitat,” a species would shift its habitat niche toward warmer microsites when the climate is cold in relation to its core habitat (Walter & Walter, 1953). This raises the question as to what extent climatic conditions at higher elevation select for higher freezing tolerance and to which extent plants use sites with a warm microclimate, or “microrefugia” (Dobrowski, 2011), to avoid freezing damage.

To study this question, we tested frost hardiness of *A. thaliana* plants from Southern Alps in the provinces Bolzano and Trento, collected from thirteen sites ranging in elevation from 280 to 2,355 m above sea level. Plants were subjected to winter freezing under near‐natural conditions during three winters on the Swabian Jura, an area known for its cold winters. Instead of using controlled artificial freezing and growing conditions performed in climatically controlled growth chambers, we used a semicontrolled approach by subjecting plants to natural winter frosts. Under natural conditions, plants are exposed to strongly varying temperatures, which is in contrast to the mostly uniform conditions in growth chambers (Poorter et al., 2016). For frost hardiness, these differences are highly relevant because it is acquired only after an acclimation period that depends on temperature fluctuations and trends preceding the frost event (Thomashow, 1999). Further, we also monitored top‐soil temperatures over two years at eight of the collection sites with data loggers. Combining these data sets, we compared the effect of elevation with the pure effect of freezing probability at the sites of origin on the evolved frost hardiness of plants. As an annual species, *A. thaliana* is part of a rare category in the high alpine flora. Nevertheless, *A. thaliana* was found up to an elevation of 4,200 m a.s.l. (Al‐Shehbaz & O'Kane, 2002; Zeng et al., 2017). There is some evidence that *A. thaliana* populations can adapt to high elevations. In a common garden study, high‐elevation populations of the Eastern Pyrenees showed higher aboveground biomass and increased fecundity, suggesting selection for higher vigor (Montesinos‐Navarro, Wig, Xavier Pico, & Tonsor, 2011). In contrast, accessions from high‐elevation populations in Switzerland were smaller and showed a reduced vigor across three common gardens at different elevations (Luo et al., 2015), although a dwarf accession showed increased fitness at high elevations. Vidigal et al. (2016) found that seed dormancy decreased, seed size increased, and plants flowered later with higher elevation of the collection site on the Iberian Peninsula. In populations from the North Italian Alps, a high differentiation of genomic regions with annotations related to ecological relevant parameters such as soil conditions, pathogen response, or soil and light response was observed (Günther et al., 2016). However, geographical patterns of the traits frost resistance, UV‐B, and light stress response did not suggest adaptation to high elevation (Günther et al., 2016). So far, we are not aware of any other study that studied frost hardiness of *A. thaliana* population from different elevations. Tests with low‐elevation accessions of *A. thaliana* suggest that the species avoids freezing via super cooling (Reyes‐Díaz et al., 2006), although freezing tolerance also plays an important role. Zhen and Ungerer (2008) showed that accessions varied in their freezing tolerance in a clinal fashion with increased freezing tolerance at high latitudes. In our study, we tested the following predictions: (a) Frost hardiness of *A. thaliana* increases with elevation. (b) The average frequency of frost explains frost hardiness better than elevation. (c) Elevation is a better predictor of frost frequency at low‐elevation sites than at high elevations. To test these hypotheses, we included a larger number of populations from the Southern Alps collected at different elevations and microsites to compare the role of different spatial scales in adaptation to elevation.

## 2 MATERIALS AND METHODS

### 2.1 Plant material

**Figure 1:**
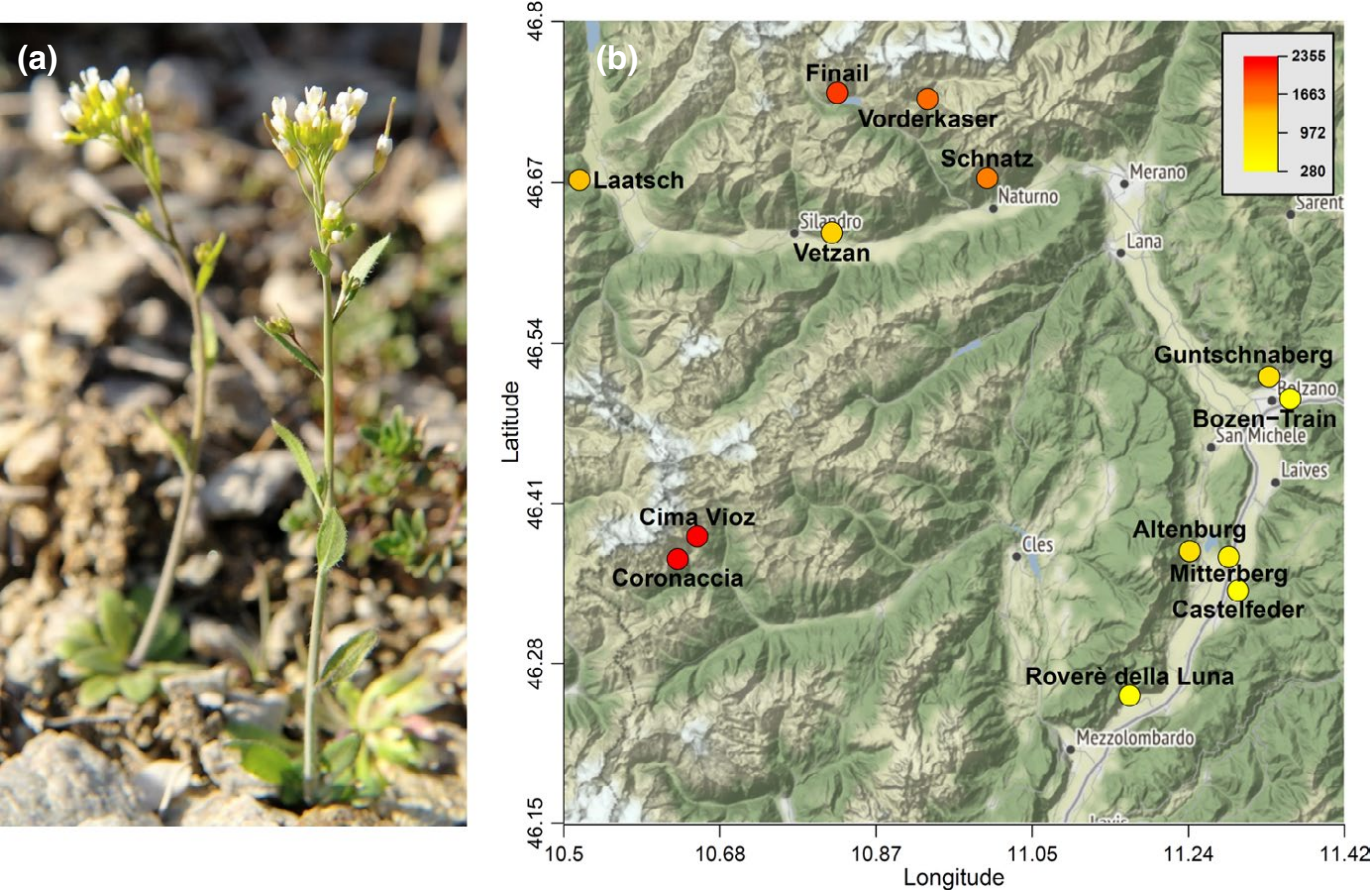
(a) *Arabidopsis thaliana* in its natural environment in Laatsch (see map) in Spring 2011. (b) Map of the *A. thaliana* collection sites in the Southern Alpine provinces of Bolzano and Trento with heat color indicating the elevation of each site. Map source: http://maps.stamen.com/terrain/?request=GetCapabilities%26service=WMTS%26version=1.0.0#11/46.5223/10.9980

We collected and geotagged seeds of *A. thaliana* accessions in the Southern Alpine provinces of Bolzano and Trento during summers from 2006 to 2008 (Figure 1a,b, Table 1). Also, we collected accessions from new microsites within the site Finail in 2011 to increase the sample size from this location. Local populations at the collection sites differed considerably in size, which is mirrored in our experiment (Table 1). We reared and self‐fertilized plants under standard greenhouse conditions to produce S1 seeds for the experiment.

**Table 1:** Characterization of sites, numbers of *Arabidopsis thaliana* accessions tested and period of logged temperatures

Site	Elevation (m)	Exposure	Disturbance (human)	Grazing/trampling	Accessions 2010/2012	Collection year	Temperature logged
Altenburg (BZ)	625	Flat	Strong	None	8/4	2008	–
Guntschnaberg (BZ)	730	SE	Little	None	8/4	2006	–
Bozen‐Train (BZ)	280	Flat	Strong	None	3/3	2006	–
Castelfeder (BZ)	390	SE‐SW	Intermediate	None	12/4	2008	2012–2015
Cima Vioz (TN)	2,355	S	Undisturbed	Chamois	1/4	2007, 2012	2012–2013
Coronaccia (TN)	2,210	SSW	Undisturbed	Chamois	4/4	2007	2013–2015
Finail (BZ)	2,166	SE	Undisturbed	Chamois	6/4	2007, 2011	2012–2015
Laatsch (BZ)	990	SE	Undisturbed	None	‐/4	2007	2012–2015
Mitterberg (BZ)	565	S‐SE	Undisturbed	None	8/4	2008	2012–2015
Roverè della Luna (TN)	315	Flat	Intermediate	None	1/1	2008	–
Schnatz (BZ)	1,709	SE	Intermediate	Sheep	1/1	2008	2012–2015
Vetzan (BZ)	880	S	Little	None	10/4	2008	2012–2015
Vorderkaser (BZ)	1,770	SE	Intermediate	Cows	3/3	2007	2012–2013

Note

In brackets province codes (BZ = Bolzano, TN = Trento)

### 2.2 Experiment

At sites where seeds were collected, *A. thaliana* follows a winter‐annual life cycle. For this life cycle, plants germinate in autumn, overwinter as rosettes, and reproduce in early spring before being overgrown by perennial plants (Donohue, 2009). The chilling in winter is required for vernalization to ensure quick transition to flowering in spring. Therefore, plants need to survive winter conditions which require frost hardiness adjusted to the local conditions at the site of origin.

**Figure 2:**
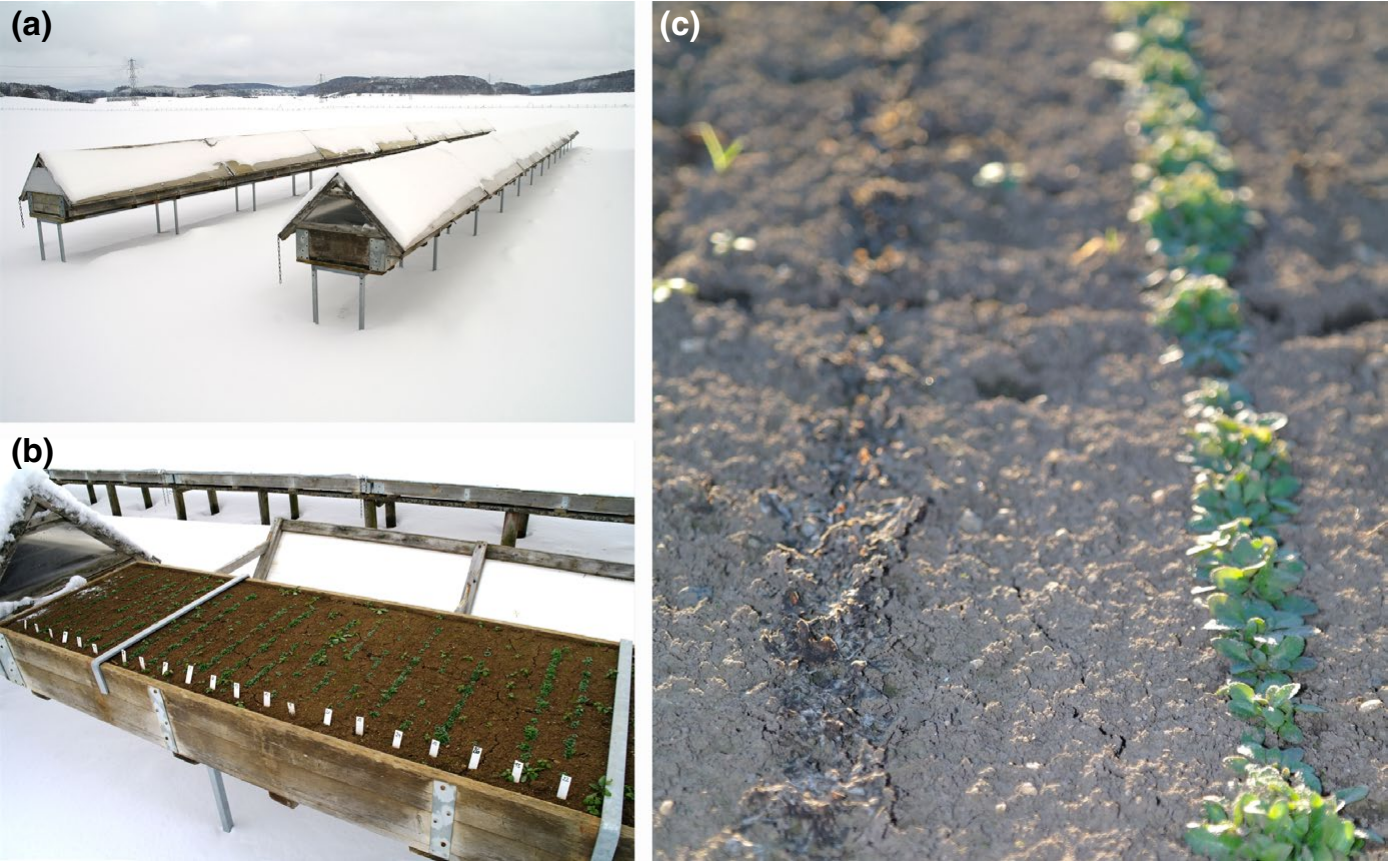
Frost testing boxes on the Swabian Jura and *Arabidopsis thaliana* plants at the time of frost damage evaluation. (a) Transparent covers keep the snow from plants and poles ensure exposure to frost from all sides. (b) The plants appear unharmed when temperatures are below or close to zero. (c) The damages are visible after thawing of the damaged tissue. Here, the left row was completely damaged, while the right row survived almost unharmed

In August 2010, we sowed seeds onto frost hardiness testing tables situated on the experimental station “Oberer Lindenhof” of University Hohenheim on the Swabian Jura (48°28′24 N, 9°18′18 E, 720 m a.s.l.). These frost testing tables were used previously to assess frost hardiness of crop varieties (Longin, Sieber, & Reif, 2013; Sieber, Longin, Leiser, & Würschum, 2016; Sieber, Würschum, & Longin, 2014). The tables were 22 cm deep, 70 cm wide, and 2.5 m, and their bottom was about 60 cm above the ground (Figure 2a,b). We filled the tables with compost (pH = 6.9, P = 31.0 mg/100 g, K = 48.0 mg/100 g, MgCaCl_2_ = 5.6 mg/100 g, humus = 5.18%) till about 5 cm below the top. Then, we watered the soil extensively before adding another layer of moist humus that filled the tables to the top. Rows of seeds were parallel to the short edge of each table, 15 cm apart. We sowed each accession to two random rows (Figure 2c), and number labeled them for blinded evaluation. Accessions were replicated in several random rows. However, the design differed between years and is described below in greater detail. Seeds were watered regularly to induce germination. After germination, we left plants to natural rainfall and irrigated only during dry periods to prevent plant loss by drought. On days with predicted snowfall during winter, a transparent cover that allowed air flow was put over the plants to keep them free of snow for direct frost exposure (Figure 2a,b). To document the freezing conditions during winter, the daily minimum and maximum temperatures were obtained by the local weather station of the experimental site that is situated within 50 m of the frost testing tables (Figure S1a).

In August 2012, we installed a second experiment on the frost hardiness testing tables. This experiment was similar to the first one, but this time we used a more balanced experimental design, because in the first year, the number of accessions differed strongly among populations (Table 1). For a more even representation, we sowed four random accessions from each site of origin, if available, each with two replicates. If the number of available accessions was below four, the available accessions were replicated more often to reach eight replicates for each site. Freezing conditions during winter were documented as daily minimum and maximum temperatures obtained by the local weather station of the experimental site that is situated within 50 m of the frost testing tables (Figure S1b).

In August 2013, we set up a third experiment on the frost hardiness testing tables. This time, space was limited. Therefore, we used population replicates instead of individual accessions. We mixed equal amounts of seeds of four accessions for each site before sowing. Only in the sites Vorderkaser (*n* = 3) and Schnatz (*n* = 1), we had fewer accessions available. In this design, each of six replicate rows resembled a true replicate for the collection site. Freezing conditions were documented by the local weather station of the experimental site (Figure S1c).

To test whether temperature measurements at the local weather station differed from the temperatures experienced by the plants, we compared them to the daily minimum temperatures measured by a data logger that was attached under one of the tables at 60 cm aboveground (Figure S2). The two data series were highly correlated for each year (2010/2011: *r* = .98, *p* < .001; 2012/2013: *r* = .99, *p* < .001; 2013/2014: *r* = .94, *p* < .001). However, especially low temperatures tended to be lower at the frost testing tables than at the standard measuring height of 2 m (Figure S2). This shows that the weather station temperatures are a good proxy for temperatures experienced by the plants with the exception of frost periods during which experienced temperatures were a few degrees lower than measured temperatures (Figure S1), which indicates that exposure to frost was even stronger. The data logger measures were not used for Figure S1 because logging was not started before the beginning of November, and it missed the coldest period in the third winter at the end of October (Figure S1).

### 2.3 Assessing frost damage

After the exposure of plants to natural winter frosts on the Swabian Jura, we evaluated the percent frost‐damaged tissue visually, while the accession identity was hidden from the monitoring person (blinded evaluation). All plants remained in the rosette state throughout the winter. Since frozen plants look green and healthy despite potentially being dead, we let the plants recover and monitored frost damage sometime after as indicated in Figure S1. At this time, the affected tissue was easily identified as dead leaf tissue (Figure 2c). The time lag between frost damage and its evaluation is unlikely to bias our estimates due to the low growth rates during this time. A similar approach for assessing frost damage after a time lag was used by Zhen and Ungerer (2008) and by Taschler & Neuner, 2004). Precisely, we estimated the percentage of dead leaf tissue visually for a whole row of plants by rating rows into 10 categories from unharmed (category 0; Figure 2c, right row) to all dead (category 10; Figure 2c, left row). As frost damage starts from the tips of the leaves and extends toward the center killing the outer leaves before the inner leaves, this means that in category 5 on average 50% of the leaves (not plants) in a row were dead. However, as this is an average for the row, higher categories exceedingly included individuals with 100% leave damage, which would most likely not recover. Only in the second year (2012/2013), a misunderstanding occurred and 12 instead of 10 categories were used for leaf damage rating. The row was the smallest unit of the experiment and was replicated in each year (see Table 1). The number of plants per row varied as a result of uneven germination. It was recorded whenever it fell below 11 replicates (Figure S3). The majority of rows, however, included more than 50 plants. When tested as weighting variable in the statistical models, the number of plants per row had no effect on the results and was therefore omitted.

### 2.4 Logging on‐site soil temperature

In each site, we logged temperature using waterproof Hobo® 8K Pendant® Temperature Loggers from Onset®. We submerged two temperature loggers in each site 3 cm in the ground to record the top‐soil temperature with a 2‐hr resolution for the whole year. This way we exposed the data loggers to the same top‐soil temperature that the plants experienced. Top‐soil temperature is probably the most relevant temperature for plant survival because winter‐annual *A. thaliana* plants remain ground‐bound rosettes until they start to reproduce in spring (Ågren & Schemske, 2012). We attached the data loggers to a nylon cord of 50–100 cm length which we covered by soil and tied to a nail that was fixed in the soil nearby a color marked shrub or rock, to facilitate recovery in the next summer. We recorded temperatures in nine sites that represent the altitudinal range covered by this study. For most sites, we measured temperatures over a period of 2.5 years from July 2012 to January 2015, except for Cima Vioz and Vorderkaser (winter 2012/2013 only) and Coronaccia (July 2013 to January 2015). We also recorded microhabitat‐specific temperatures in the two highest sites, Cima Vioz and Coronaccia. Here, *A. thaliana* plants grew underneath steep rock walls and in cracks in these rocks. We installed data loggers both below the rocks and in rock cracks that were large enough to support soil accumulation to ensure that soil temperature was measured.

### 2.5 Analysis of temperature records

Selection for frost hardiness depends on two aspects of local microclimate. First, the absolute minimum temperature that plants experience at a site, and second, the frequency of such low frost events at a site (Inouye, 2000). To differentiate between the effects of both microclimate variables on selection for frost hardiness would require multiple years of temperature data from multiple sites. However, if only data from a few years are available, useful surrogates for both variables can be extracted from the data. For example, the lowest observed temperature in a site can be used as a proxy for the absolute minimum temperature in a site. For comparing the populations, the relative difference among sites is more important than the absolute measured values. In a similar fashion, we can count for each data logger the number of days with temperatures below 1°C. An independent calibration on the Swabian Alb showed that the number of days below 1°C measured by a buried data logger 3 cm belowground equaled the number of days below zero observed for the same period by the local weather station. To apply lower temperature thresholds was not possible, because our sample included sites in the valley where soil temperatures did not decrease below zero during winter. A lower temperature threshold would not differentiate between these sites. Because single lowest temperature observations are less reliable than the number of frost days across two winters, we used the latter as main covariate and used the first only for comparison.

We trimmed temperature data from each data logger to cover two years starting and ending in mid‐summer. For each data logger, we counted the number of days with temperatures below 1°C. We averaged over all data loggers and recorded winters for each site to obtain an estimate for frost exposure of winter‐annual plants. This estimate could be biased if the two winters were very different because we did not have records from both winters for all sites. Using the sites with full records, we fitted a generalized linear model with Poisson error family (corrected for overdispersion), to test whether years differed across sites in their counts for days with temperatures below 1°C (frost days aboveground). The model contained the dependent variable frost days and the independent variables year, site, and their interaction. As expected, the number of frost days differed strongly between sites (*F* = 125, *df* = 5/14, *p* < .001). However, there was no significant difference among years (*F* = 1.29, *df* = 1/19, *p* = .28) and no interaction between sites and years (*F* = 0.7, *df* = 5/9, *p* = .64). We concluded that the two winters were similar enough and required no correction.

### 2.6 Analysis of frost damage

We evaluated frost damage as an average over all plants in a row (Figure 2b,c). The estimated frost damage is then a measure of central tendency. The central limit theorem states that the distribution of means quickly approaches normality with increasing sample size of each mean, independently of the distribution of the data. We therefore used percentage frost damage as dependent variable with a Gaussian error distribution. To test whether frost damage correlates with the elevation of collection sites, we fitted a linear mixed effects model with the following equation:

(1) $${\text{frost damage}}_{\mathrm{ijk}}=\mu +\ln{\left(\text{elevation}\right)}_{i}+{\text{year}}_{j}+\ln\left(e\right)\times y_{\mathrm{ij}}+\varepsilon_{\mathrm{ijk}}$$

where *µ* is the overall intercept, ln(elevation)*_i_* the fixed effect of the natural logarithm of elevation of population *i*, year*_j_* the fixed effect of year*_j_* and ln(*a*)×*y_ij_* their interaction. With the random effect ac*_k_* of accession *k,* we accounted for the nonindependence of replicates of the same accession. The term *ε_ijk_* is the residual associated with the replicate of accession *k* from elevation *i* in the year *j*. We computed the model using the R package lme4 (version: 1.1‐17; Bates, Mächler, Bolker, & Walker, 2015). To test whether elevation or days below 1°C explained more variance in frost damage, we included this variable in the fixed effects part according to the following equation:

(2) $${\text{frost damage}}_{\mathrm{ijk}}=\mu +\ln{\left(\text{elevation}\right)}_{i}+{\text{frost days}}_{i}+{\text{year}}_{j}+\text{ac}{\left(\text{year}\right)}_{k(j)}+\varepsilon_{\mathrm{ijk}}$$

with the overall intercept *µ*, the logarithmic elevation effect of population *i*, the effect of the number of frost days of population *i*, and the random effects of year *j* and of accession (ac) *k* nested in year *j*. The term *ε_ijk_* is the residual defined like in Equation 1. The random effects were tested for models fitted with restricted maximum likelihood (REML) using either likelihood ratio tests by comparing two nested models or using the function ranova from the R package lmerTest (version: 3.01; Kuznetsova, Brockhoff, & Christensen, 2017). For the fixed effects, we refitted the models using maximum likelihood and applied Bayesian inference with a flat prior using the nsim function in the R package arm (version: 1.10‐1; Gelman & Yu‐Sung, 2018). As a measure of statistical significance, we present the Bayesian 95% credible interval (CrI 95%) for each estimate.

Further, we tested for a linear relationship between soil temperature (frost days) and elevation of the collection site using the lme function from the R package nlme package in R (version: 3.1‐137; Pinheiro, Bates, DebRoy, & Sarkar, 2016), because it includes functions to account for heterogeneous variances. We fitted a mixed effects model of the form:

(3) $${\text{frost damage}}_{\mathrm{ij}}=\mu +{\text{elevation}}_{i}+{\text{year}}_{j}+\varepsilon_{\mathrm{ij}}.$$

The model contained the overall intercept *µ*, the fixed effect of the elevation of site *i,* and the random effect of year *j*. The term *ε_ij_* is the residual of the replicate in elevation *i* and year *j*. To model the variance proportionally to elevation, we used the varPower function with the weights argument in the lme function. This means that the residuals had the form:

(4) $$\varepsilon_{\mathrm{ij}}∼N\left(0,\;\sigma^{2}\times {\left|{\text{elevation}}_{i}\right|}^{2C}\right)$$

Here, *N* is a normal distribution with mean zero and variance *σ* ^2^ multiplied with the power of the absolute value of the elevation of site *i*. The variance function coefficient *C* is estimated from the data (Zuur, Ieno, Walker, Saveliev, & Smith, 2009).

## 3 RESULTS

### 3.1 Accessions from higher elevation are more frost resistant

**Figure 3:**
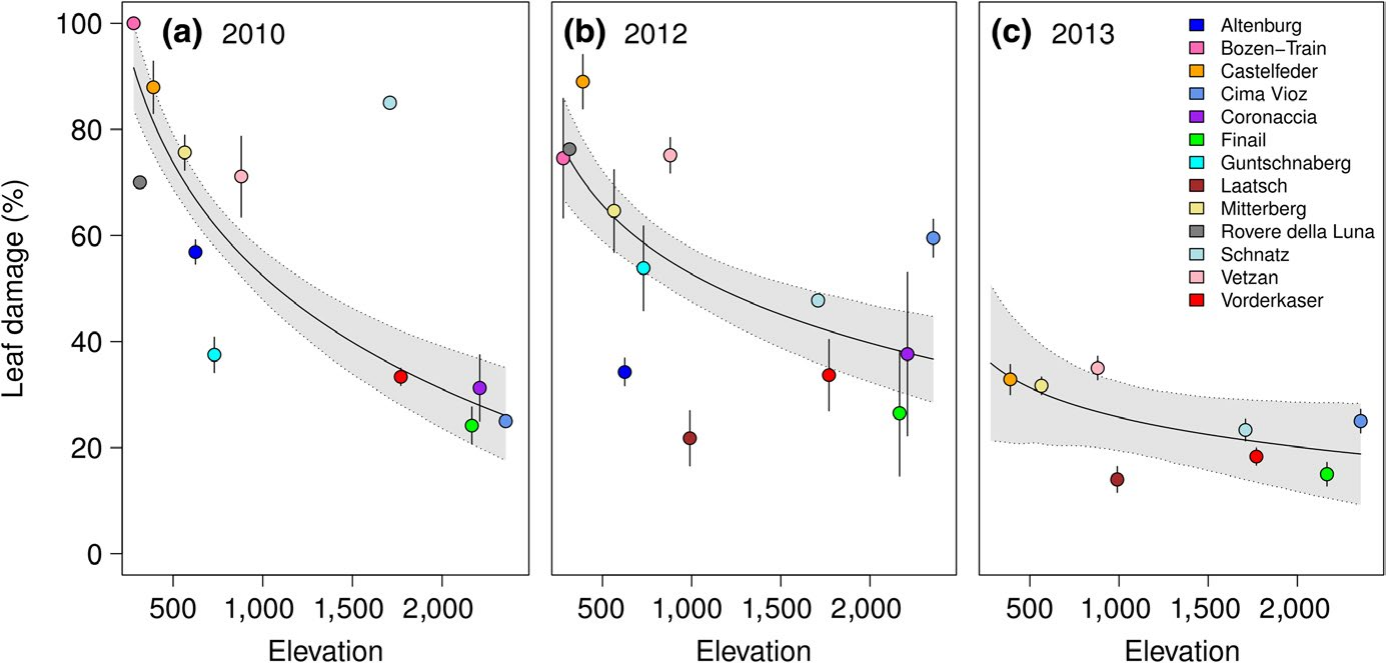
Frost damage of *Arabidopsis thaliana* populations as a function of the elevation of the collection site. Populations from higher elevations show reduced frost damage especially in (a) winter 2010/2011, (b) winter 2012/2013, and less in (c) winter 2013/2014. The regression line is shown together with its 95% credible interval. In addition, for each site the averages and standard errors across accession means are presented. In the last year (c), the standard error was estimates across samples

Our common garden experiment showed that frost damage significantly decreased with increasing elevation of the collection site if values were averaged across all three years (*b* = −19.2, CrI 95%: −32.8; −5.8). The three years differed strongly in frost damage, which was mostly due to strong variation in frost damage of the low‐elevation populations (Table 2). The variance of predicted frost damage across years at 280 m (*σ* ^2^ = 827) was ten times the variance of frost damage estimates from 2,355 m elevation (*σ* ^2^ = 80.4). Frost damage was strongest in the first winter (2010/2011; Figure 3a), lower in the second winter (2012/2013; Figure 3b), and lowest in the third winter (2013/2014; Figure 3c). These differences between years match the number of relevant deep frost events (below −7°C), in each winter. In a previous freezing experiment, we found that the exothermic peak of freezing plant material of South Tyrolian *A. thaliana* plants was around −7°C (Günther et al., 2016). The number of days with temperatures below −7°C in the respective winters before frost damage was 18 in the first winter, 14 in the second winter, and 2 in the last winter (Figure S1). Consistent with the general frost damage, the regression slope changed strongly between years (Table 2). Frost damage reduced dramatically with elevation of the collection site in the first winter, and the slope was less steep in the second winter and nonsignificant in the last winter (Table 2). Notably, the model had been significantly improved by fitting frost damage to the natural logarithm of elevation, instead of assuming a simple linear relationship (*χ* ^2^ = 4.8, *df* = 0, *p* < .001, Table S1). This indicates that frost hardiness showed a stronger correlation with elevation at the low end of the gradient than at its high end. To demonstrate the effect size of this change, we calculated the frost damage increment for the lowest and the highest 500‐m elevation segment (Table 2). The frost hardiness increment was 4.3‐fold lower for high‐elevation populations than for populations in the lowest elevation segment. This indicates that the elevation difference between populations is less important for variation in frost hardiness at high elevation.

**Table 2:** Model predictions for the percentage of frost‐damaged leaf area and the change in frost damage with elevation for the high‐ and the low‐elevation ends of the gradient in 3 years

Year (winter)	Predicted frost damage		Predicted change in frost damage	Predicted frost damage increment	
	Low elevation (280 m a.s.l.)	High elevation (2,355 m a.s.l.)	Slope *b*	Lowest 500 m	Highest 500 m
2010/2011	92 (84; 99)	26 (18; 34)	−31 (−37; −25)	−32 (−35; −28)	−7 (−9; −6)
2012/2013	77 (67; 86)	37 (28; 45)	−19 (−34; −4)	−19 (−24; −14)	−4 (−6; −3)
2013/2014	36 (21; 51)	18 (9; 29)	−8 (−26; 11)	−8 (−16, 0.33)	−2 (−0.3; −4)

Note

The corresponding 95% credible intervals are presented in brackets.

### 3.2 Top‐soil temperature is a better predictor than elevation

**Figure 4:**
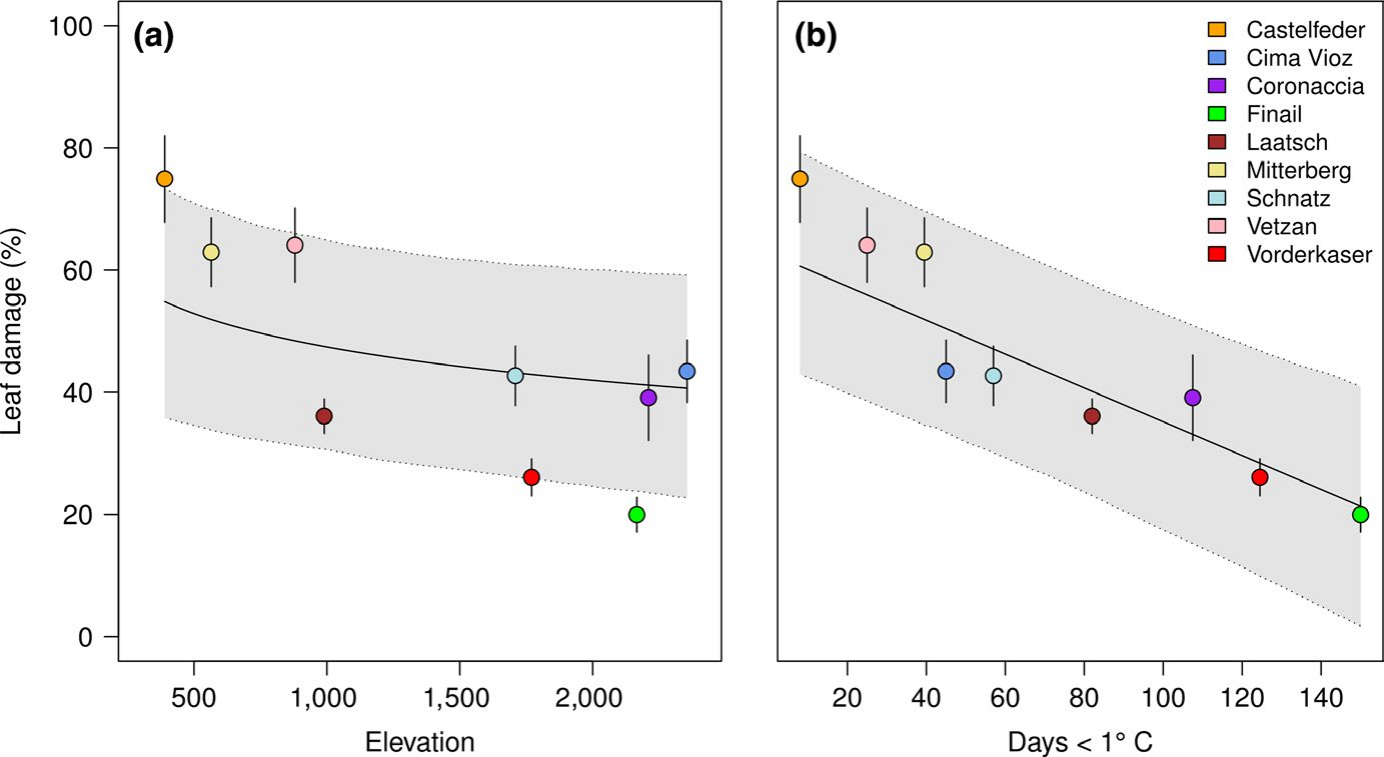
Partial regression lines and their 95% credible interval (gray) for frost damage of *Arabidopsis thaliana* populations regressed on elevation (a) and frost days (b) averaged across three years. Points represent population averages with standard error

To compare the effect of elevation with in situ measured temperature, we reduced the data set to the populations where the temperature was recorded and included the year as a random effect component (see Section 2 for details). We found that the number of recorded frost days in the collection site (days with minimum temperature below 1°C, see Section 2 for explanation) was a stronger predictor for differences in frost damage than the elevation of the collection site as can be seen from their partial regression slopes (Figure 4). Accordingly, the variable frost days explained more variance (*F* = 32.1) than elevation (*F* = 4.7), based on type II sums of squares. Further, when fitting the two explanatory variables in separate models, the model with frost days had a lower AICc (frost days AICc = 1,744.4; ln elevation AICc = 1,768.0). For the number of frost days, the partial regression slope suggested a reduction in leaf damage of −0.28 (CrI 95%: −0.37; −0.18) with each additional frost day in the collection site of a population. In other words, averaged across the three years, if the number of frost days increased by 10 days from one to the next collection site, the frost damage of the respective plants in our common garden experiment decreased on average by 3%. The slope for elevation was −7 and was also significant (CrI 95%: −15; −0.66). These two slopes are not directly comparable, because elevation was log‐transformed (Table S2), showing that the change in frost damage was strong between valley populations and decreased toward high elevations, as was found for the individual years (Table 2). As an additional test whether elevation or the number of frost days better explained the variation in frost damage, we use the posterior distribution of 2,000 simulations to ask how likely it is to observe a slope equal to or greater than zero. While for elevation non‐negative slopes were observed in 2% of the simulations, for frost days, all slopes were negative, again demonstrating the superiority of frost days in explaining the observed differences between populations in frost damage. We obtained qualitatively similar results when we used the temperature‐site‐minimum as covariate instead of the number of frost days (Table S3). However, the number of frost days at a site was a superior predictor of frost damage in a comparison with two models that differed only by this covariate (ΔAIC = −13.5, *χ* ^2^ = 13.5, *df* = 0, *p* < .001).

### 3.3 Mismatch between elevation and soil temperature increases with elevation

**Figure 5:**
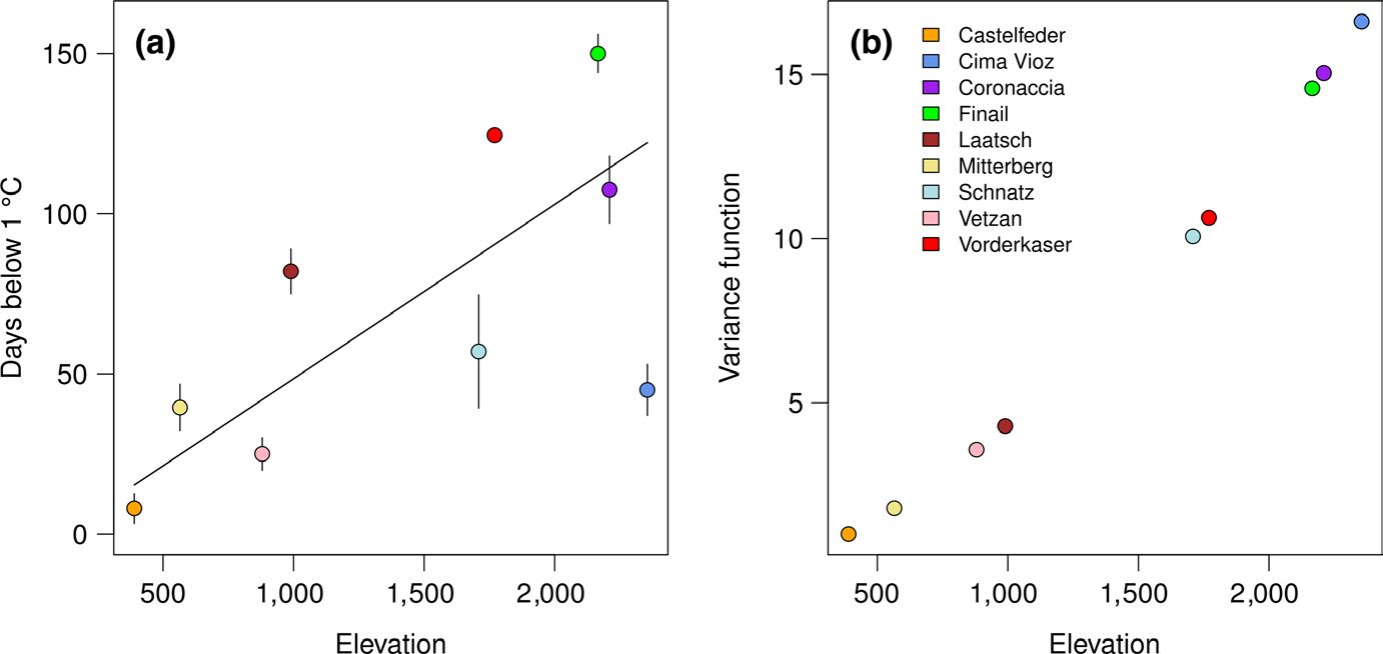
The effect of elevation on top‐soil temperature. (a) Regression of frost days on elevation with site averages across data loggers and winters and standard errors. (b) Fitted values for the variance power function of the form *f*(altitude) = |elevation*_i_*|^(2 ×^ *^C^* ^)^ (*C* = variance function coefficient) that was used to model the increase of residuals with elevation

The observation, that the number of frost days explained variation in frost damage better than elevation, suggests that the probability to experience ground frost is not a simple function of elevation. To better understand how elevation influences the number of frost days, we next tested whether there is a linear relationship between soil temperature and elevation of collection sites. Elevation indeed had a strong positive effect on the number of days below zero (Figure 5a; *F*_1/24_ = 25.46, *p* < .001); however, the residual distribution was heterogeneous, as indicated by a significant Fligner–Killen test of homogeneity of variances (*χ* ^2^ = 17.03, *df* = 8, *p* = .029). Notably, the variance strongly increased together with elevation as can be seen from the population averages displayed in Figure 5a. We modeled this effect with a power function for the variance using the weights argument in the lme function (Pinheiro et al., 2016). As a result, the effect of elevation increased (*F*_1/24_ = 40.17, *p* < .001) and the corrected residuals were distributed evenly. A likelihood ratio test showed that the correction of variance heterogeneity improved the model (ΔAICc = −7.5, *L* = 10.5, *df* = 1, *p* = .001). The variance power function is plotted in Figure 5b and suggests an exponential increase of residuals with elevation with a 15‐fold increase in variance from 280‐ to 2,355‐m elevation.

**Figure 6:**
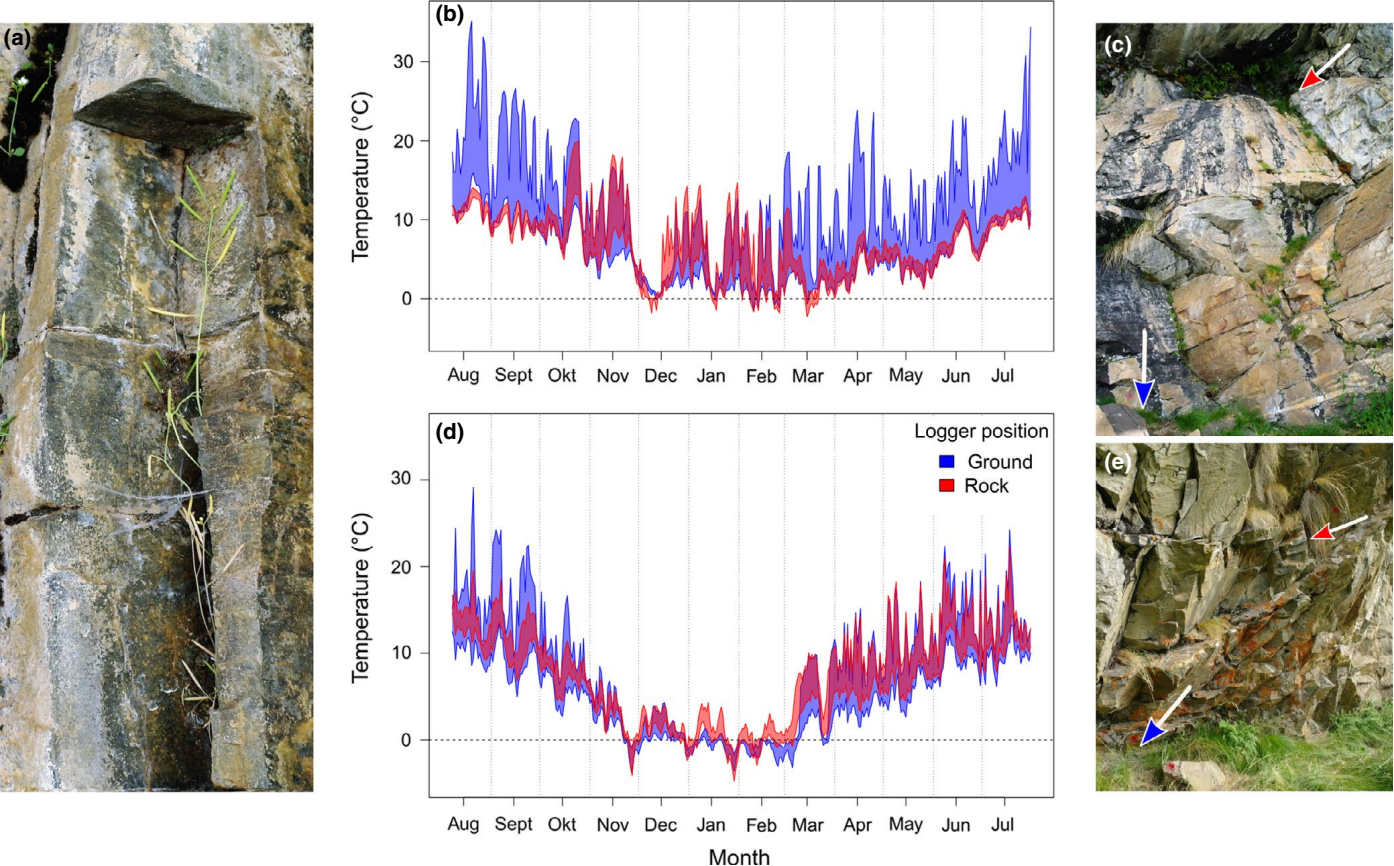
Top‐soil temperatures across the year in two microhabitats of *Arabidopsis thaliana* in the two highest populations. (a) *A. thaliana* plants growing in rock cracks at the site Cima Vioz. (b) Minimum and maximum top‐soil temperatures of the two microhabitats rock and ground in Cima Vioz with the logger positions indicated by colored arrows in (c). (d) Minimum and maximum top‐soil temperatures in Coronaccia with the logger positions indicated in (e). The temperature diagrams indicate that the loggers were not covered by snow even in winter (compare to Figure S4)

To characterize the difference between two closely located microhabitats that harbored *A. thaliana* populations at the highest elevation, we compared top‐soil temperatures in rock cracks and below rocks (Figure 6). In both sites, the two microhabitats differed strongly in number of days with ground frost (i.e., days with temperature < 0°C). Here, we counted the days with soil temperatures below zero because all temperature records included sufficient observations of ground frost. In Cima Vioz, we measured 29 ground frost days in the rock crack and 13 in the soil below the rocks during the winter 2012/2013. The Coronaccia site experienced 55 frost days in the rock crack and 82 in the soil below the rocks in the winter 2013/2014. Although measurements were taken in different winters, the daily maximum temperatures showed much higher variance on the ground (Cima Vioz: *σ* ^2^ = 53.7; Coro: *σ* ^2^ = 50.1) than in the rock crack (Cima Vioz: *σ* ^2^ = 17.4; Coro: *σ* ^2^ = 31.1) at both sites. Taken together, these data demonstrate that microsites indeed strongly differ in their temperature regime in the high mountains.

## 4 DISCUSSION

### 4.1 Frost hardiness of *A. thaliana* increases with elevation

In line with our initial prediction, frost hardiness increased on average with the elevation of the collection site. This result is consistent with the well‐documented difference in frost hardiness between low‐elevation and high‐elevation species (Earnshaw et al., 1990; Taschler & Neuner, 2004). However, with respect to intraspecific variation in frost hardiness, the existing literature is more ambiguous. Sierra‐Almeida et al. (2009) found higher frost resistance of populations from higher elevations in four out of seven studied species from the high Andes. In the three remaining species, frost hardiness did not differ between high and low elevations. Frost hardiness also increased with higher elevation in the fern *Blechnum penna marina* from New Zealand (Bannister & Lee, 1989) and in *Solanum acaule* from Peru (Li, Palta, & Hawkes, 1980). *Trifolium repens*, on the other hand, showed no differences in frost hardiness between low‐ and high‐elevation populations in Sweden (Junttila, Svenning, & Solheim, 1990). These differences between studies and species are surprising given that frost damage entails a serious fitness cost (Agrawal, Conner, & Stinchcombe, 2004). It suggests that elevation of provenance is not consistently a good proxy for frost hardiness. Notably, in our study a log‐linear curve greatly improved the model, suggesting that frost hardiness changed rapidly with elevation at lower elevations, but slowly at high elevations. Indeed, frost hardiness did not increase much above 1,000 m despite strong variation among populations. This observation reflects our results in an earlier study with *A. thaliana* populations from the same region in South Tyrol, in which the differences in frost hardiness among populations did not covary with the elevation of the five collection sites (Günther et al., 2016). In the present study, we included more populations from lower elevations, which improved the statistical power of the analysis of the relationship between frost hardiness and elevation providing a solid proof of concept for future research. Besides the variation among populations, we also observed differences among years. On average, these differences matched the differences in severe frost days at the common garden site on the Swabian Jura between three winters. However, beyond these differences also some individual populations varied strongly between the first two years, for example, at Altenburg, Bozen‐Train, Schnatz, or Cima Vioz (Figure 3). These differences may have several reasons. First, the experimental design differed among years, with some populations being represented by different accessions in different years (Table 1). However, two populations that were among the group showing the strongest variation (Bozen‐Train and Schnatz) were always represented by the same accessions throughout the experiment. Notably, other populations like Castelfeder, Vetzan, and Finail showed nearly the same frost damage in the first two years (Figure 3). Therefore, the differences among years could be attributed to a genotype × environment interaction, that is, differences in phenotypic plasticity among genotypes. For example, genotypes could differ in their frost acclimation that interacts with the temperatures preceding the frost events in each year (Thomashow, 1999). This has been observed previously for frost hardiness in *Trifolium repens* (Junttila et al., 1990). Individual inconsistencies could also indicate errors during monitoring, but our blinded monitoring approach makes this unlikely. Three years of monitoring and a sufficiently high number of populations along the elevation gradient make our results robust against individual deviations. Taken together, we observed high variation among populations and years that was partly associated with elevation. One reason for the high tendency of provenances to depart from the linear prediction for their elevation may be that frost hardiness is more closely connected to the local microclimatic conditions than to the general climatic conditions at a specific elevation level.

### 4.2 The probability of ground frost explains frost hardiness better than elevation

We found that the frequency of ground frost estimated from top‐soil temperatures at collection sites was superior to elevation as predictor of frost hardiness differences between *A. thaliana* populations. In contrast to elevation, the frequency of frost days showed a linear relationship with frost hardiness. This is in line with our second prediction and confirms that the microclimate rather than the elevation of a site accounts for the frost hardiness of local populations. It is known that mountains are characterized by a high degree of microtopographic differences that influence the local microclimate (Briceño, Harris‐Pascal, Nicotra, Williams, & Ball, 2014; Lembrechts et al., 2018). In particular, local frost conditions play an important role in microclimate adaptation. Wos and Willi (2018) showed that genotypic differences in frost hardiness of *Arabidopsis lyrata* were linked to vegetation cover on a scale of a few meters in a sand dune landscape. In alpine environments, populations of *Aciphylla glacialis* from sites with early snow melt showed stronger frost hardiness than populations from sites with later snow melt (Briceño et al., 2014). Also, frost damage of flower buds from three mountain wildflower species was associated with differences in snow accumulation, snowmelt pattern, and cold air drainage on a scale of few meters (Inouye, 2008). In particular, a high snow cover protects plants from low negative temperatures and is an important factor for local frost adaptation (Larcher, Kainmüller, & Wagner, 2010). However, in the present study snow may not play an important role because *A. thaliana* populations occupied only SE‐ to SW‐exposed slopes at high elevation (Table 2). On south‐exposed slopes, the sun can melt snow even during winter, leaving the plants exposed to freezing during the night or on cold days (Lembrechts et al., 2018). Thick snow layers reduce the diurnal temperature amplitude to a flat line close to zero (Larcher et al., 2010). This pattern was indeed observed for shorter periods in two high‐elevation sites (see Figure S4), but was absent from other high‐elevation sites (Figure 6b,d). However, these temperature curves also demonstrate that even in the two sites which occasionally are covered by snow, plants were exposed to strong frost in the middle of winter. This shows that *A. thaliana* in the North Italian Alps is a typical rock outcrop, and the danger of freezing damage exists in most sites throughout the winter. Furthermore, the mild microclimate of south‐exposed sites in alpine ecosystems supports plant growth and establishment of species whose core distribution range is at much lower elevation (Lembrechts et al., 2018). Our focal species, *A. thaliana* can be seen as a typical example for this phenomenon. As annual plant, it shows a life history that is strongly underrepresented in high alpine plant communities (Körner, 1995). In conclusion, microclimatic heterogeneity is strong in alpine environments and affects plant growth and fitness independently of elevation. Indeed, the degree of decoupling of local microclimate from the average elevation trend can be strong enough to support populations with properties that would otherwise be found at a much lower elevation.

### 4.3 Ground frost probability increasingly varies with higher elevation

We observed an increasing variance in top‐soil temperature with increasing elevation of the site. Some high‐elevation sites showed a similar number of frost days as low‐elevation sites, suggesting that at high elevation some *A. thaliana* populations exist in favorable microrefugia, which is consistent with the “law of the relative constancy of habitat” of (Walter & Walter, 1953). Such microrefugia are common in high mountains (Dobrowski, 2011; Graae et al., 2012). According to Graae et al. (2012), the local temperatures in high mountain sites, when measured in situ, are mostly higher than expected from interpolations across weather stations. The authors attributed this effect to inverted temperatures in winter, when cold air downdrafts hinder the accumulation of cool air at high‐elevation sites. In line with this suggestion, all high‐elevation sites in our study were situated on steep predominantly south‐exposed slopes well above the cold air drainage that must be expected in the couloirs. Microclimatic conditions may also be influenced by differences in the effective heat capacity of soil and base rock. Accordingly, we found strong differences in temperature profiles between soil in rock cracks and the soil below the rocks, which represent two microhabitats that were occupied by *A. thaliana* in the two highest sites. Specifically, the soil temperature in rock cracks showed reduced variation in summer, which can serve to buffer extreme heat peaks. However, also the most frost‐hard population Finail was one of the high‐elevation sites, which demonstrates that *A. thaliana* was not restricted to warm microrefugia at high elevations. Besides habitat sorting according to the law of Walter and Walter (1953), also adaptation in frost hardiness played a role in the successful survival of *A. thaliana* at high‐elevation sites. In this population, plants were found underneath larch trees which intercept snowfall reducing the snow depth beneath their crown. Snow layers are known to function as insulating layers that efficiently reduce frost damage (Inouye, 2008). This also matches the observation that frost hardiness was associated with vegetation cover in *A. lyrata* (Wos & Willi, 2018). The potential reasons for microclimate heterogeneity are multifold. Together, our results suggest that at high‐elevation microclimate effects on top‐soil temperatures may overcome average elevation effects. This was also suggested by Shreve (1924), who compared soil temperatures of north and south slopes at different elevations. However, in contrast to Shreve (1924) who chose representative sites for each elevation, our sites mark actual populations of a species and we show that the microclimate had strong effects on local plant adaptation. This highlights two aspects that may be important for the survival of some plant species in the face of climate change. First, the microclimate heterogeneity of high alpine environments may preserve a high functional genetic diversity among many small populations that are adapted to different microhabitats. Genomic results from our previous study suggested that high‐ and low‐elevation populations of *A. thaliana* from South Tyrol split before the last glacial maximum, ca. 18,000 years ago (Günther et al., 2016). These small populations could become important in future climate change scenarios that require their phenotype. At the same time, however, they may be endangered because recent findings suggest that climate change may disrupt historical connections between local environmental variables driving these small locally adapted populations out of their optimum (Wadgymar, Ogilvie, Inouye, Weis, & Anderson, 2018). Second, the observed microclimate heterogeneity and its effects on the persistence of differently adapted populations at high elevation supports the observation of Dobrowski (2011) and Graae et al. (2012), that using large scale climatic parameters to predict the fate of a species in a global warming scenario, as is common practice in climate‐envelope modeling, may seriously under‐ or overestimate the suitable habitat that is available in topographically complex regions.

## CONFLICT OF INTEREST

None declared.

## AUTHOR CONTRIBUTIONS

C.L. and K.J.S conceived and designed the study; J.W., T.W., K.J.S, and C.L. found the sites and collected the seeds, C.L. conducted the experiment, performed the statistical analysis, and wrote the first version of the manuscript, with J.W., T.W., and K.J.S contributing to revisions.

## Data Availability

The frost hardiness data together with the microclimate variables are published at Zenodo.org with the https://doi.org/10.5281/zenodo.3379429
