# Supplementary material for: Microclimate predicts frost hardiness of alpine Arabidopsis thaliana populations better than elevation
Source: Ecol Evol. 2019 Oct 9;9(23):13017–29. doi: 10.1002/ece3.5659 (PMC6912909; doi:10.1002/ece3.5659)
Supplement: Supplementary file 1 [file ECE3-9-13017-s001.docx]

**Supporting Information**

Article title: Microclimate predicts frost-hardiness of alpine *Arabidopsis thaliana* populations better than elevation

Authors: Christian Lampei, Jörg Wunder, Thomas Wilhalm, Karl J. Schmid

**Table S1** Model comparison for testing changes in frost-hardiness along altitude across 3 years. The R^2^-values were calculated using the r.squaredGLMM function of the R-package MuMin that uses the method of Nakagawa et al. (2017).

| Model | AICc | Marginal R^2^ | Conditional R^2^ |
| --- | --- | --- | --- |
| Random effects (REML): |  |  |  |
| Leaf damage ~ altitude * year + (1 \| accession) | 2399.2 |  | 0.59 |
| Leaf damage ~ altitude * year + (1 \| accession/year) | 2398.4 |  | 0.54 |
| Fixed effects (ML): |  |  |  |
| Leaf damage ~ altitude * year + (1 \| accession) | 2382.6 | 0.43 | 0.55 |
| Leaf damage ~ log(altitude) * year + (1 \| accession) | 2369.7 | 0.47 | 0.56 |

**Table S2** Model comparison for testing changes in frost-hardiness with the recorded number of frost day and the elevation of each site. The R^2^-values were calculated using the r.squaredGLMM function of the R-package MuMin that uses the method of Nakagawa et al. (2017).

| Model | AICc | Marginal R^2^ | Conditional R^2^ |
| --- | --- | --- | --- |
| Random effects (REML): |  |  |  |
| Leaf damage ~ elevation * year + (1 \| accession) | 1742.5 |  | 0.61 |
| Leaf damage ~ elevation * year + (1 \| year/ accession) | 1743.8 |  | 0.64 |
| Fixed effects (ML): |  |  |  |
| Leaf damage ~ elevation * year + (1 \| year / accession) | 1743.2 | 0.33 | 0.59 |
| Leaf damage ~ log(elevation) * year + (1 \| year / accession) | 1741.9 | 0.34 | 0.60 |

**Table S3** Linear mixed effects model for site-temperature-minimum as microclimate covariate. The denominator degrees of freedom for the F-test were estimated according to Satterthwaite’s method.

| **Variable** | **Sums of squares (SS)** | **Mean SS** | **Df/DF** | **F-Value** | **p-value** |
| --- | --- | --- | --- | --- | --- |
| log(elevation) | 1469.4 | 1469.4 | 1/134.33 | 3.88 | P=0.051 |
| Site-temperature-minimum | 5841.5 | 5841.5 | 1/133.45 | 15.43 | p>0.001 |


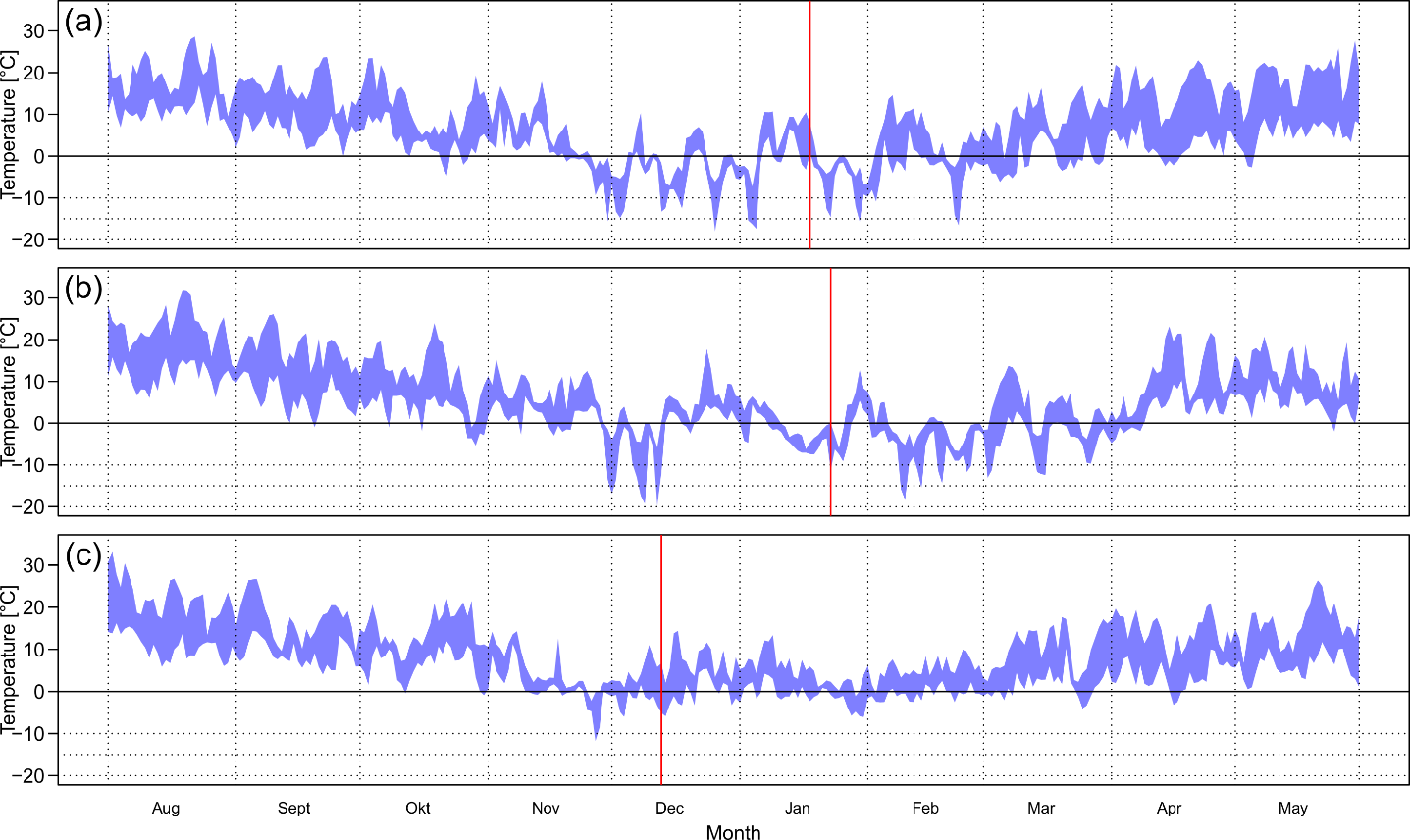


**Figure S1** Minimum and maximum temperature in the experimental site for the winter (a) 2010/2011, (b) 2012/2013 and (c) 2013/2014 obtained by the weather station of the experimental site. The red vertical line indicates the date when frost damage was evaluated, this differs among years because the winters were different (especially 2013/14 had deep frost only in November). The number of days below 0 °C before the evaluation was (a) 55, (b) 52 and (c) 24. The number of days below -7 °C before the evaluation was (a) 18, (b) 14 and (c) 2. Due to technical problems, no weather data was available for February and March 2014. For this time the next neighboring weather station at similar elevation was used (St. Johann, 3 km distance at 749 m elevation).

**Figure S2** Correlation of daily minimum temperatures measured at the frost testing tables (x-axis) and the local weather station (y-axis). While the bottom of the frost testing tables was 60 cm above ground, the temperatures measurements at the weather station were normal standard measures taken at 2 m above ground.


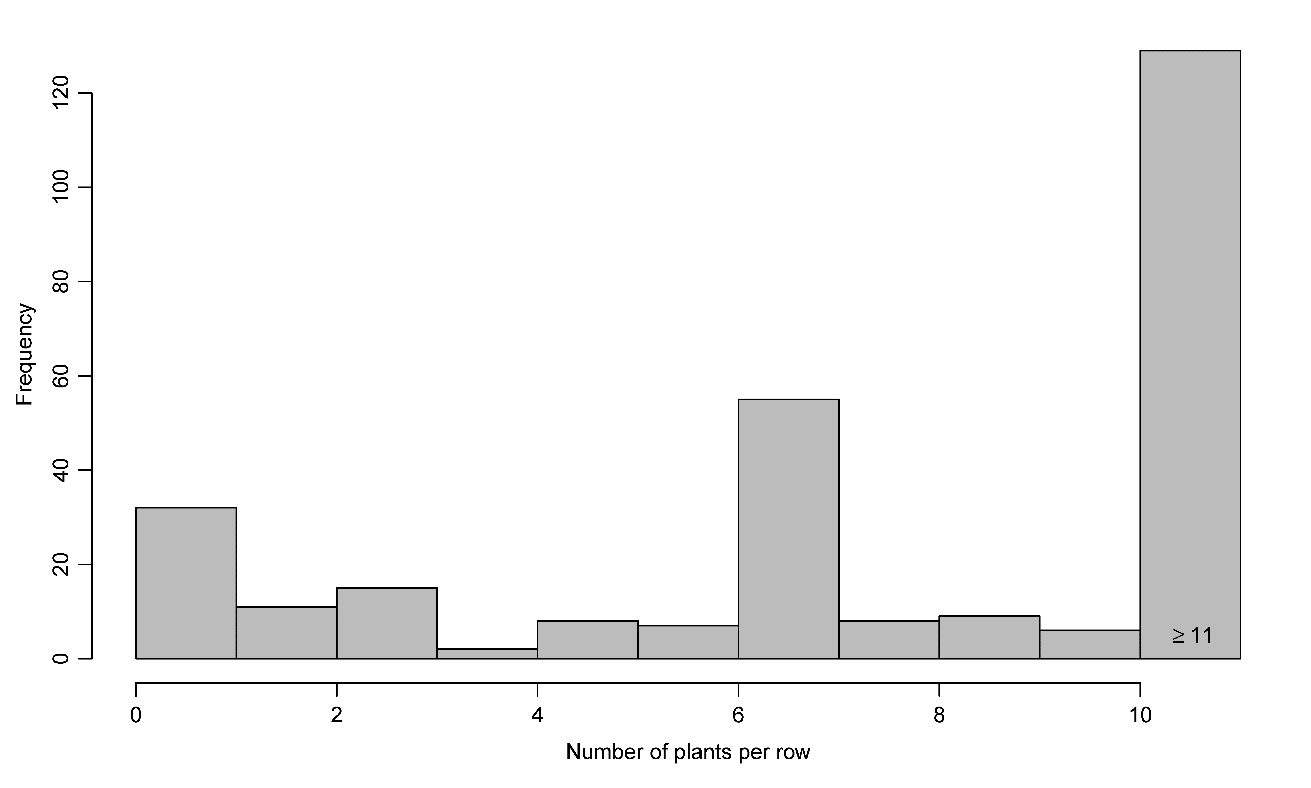


**Figure S3** Frequency distribution of the number of plants per row. From 11 plants onwards counting was omitted because 11 was considered a reasonable number of individuals for a row. Also, we had time constraints because most rows above 11 plants actually had 50 and more individuals.


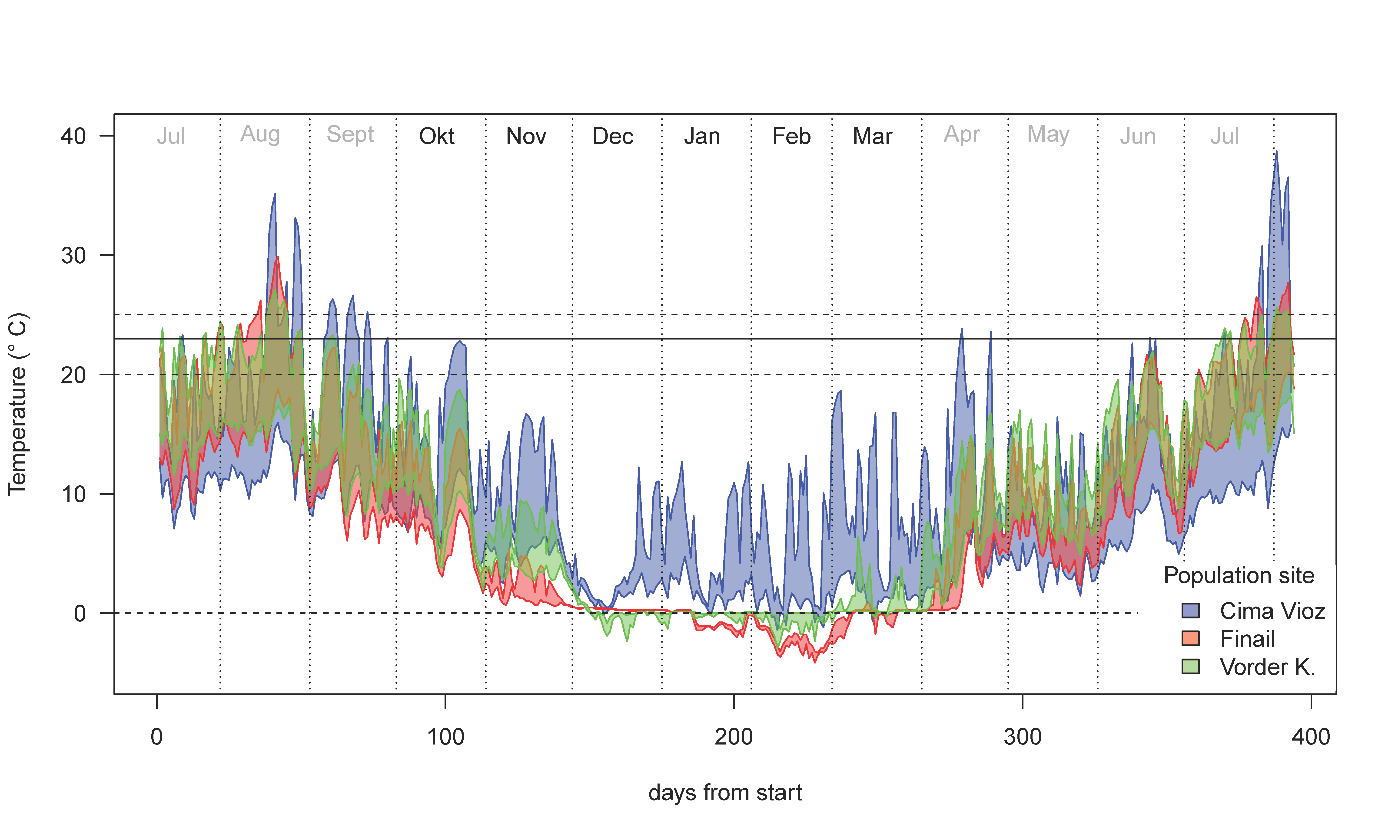


**Figure S4** Daily minimum and maximum temperatures of three high-elevation populations. The Cima Vioz temperatures are identical with the ground temperatures in Fig. 6b. Horizontal lines indicate the zero line and the usually assumed temperature optimum of *A. thaliana* for greenhouse cultivation between 20 °C and 25 °C. Due to its insolating attitude the diurnal amplitude decreased to a flat line when the data logger was covered by a sufficient snow layer. Which was the case in Finail and Vorderkaser, however only for short periods of at a time. The data show clearly that plants were exposed to freezing temperatures even in the middle of winter.

References cited within the Supporting material

**Nakagawa S, Johnson PCD, Schielzeth H**. **2017**. The coefficient of determination R 2 and intra-class correlation coefficient from generalized linear mixed-effects models revisited and expanded. *Journal of The Royal Society Interface* **14**: 20170213.
